# Availability of pulmonary rehabilitation in primary care for patients with COPD: a cross-sectional study in Sweden

**DOI:** 10.3402/ecrj.v3.31601

**Published:** 2016-11-28

**Authors:** Mats Arne, Margareta Emtner, Karin Lisspers, Karin Wadell, Björn Ställberg

**Affiliations:** 1 Primary Care Research Unit, Karlstad, Sweden; 2 Department of Medical Sciences, Respiratory, Allergy and Sleep Research, Uppsala University, Uppsala, Sweden; 3 Department of Public Health and Caring Sciences, Family Medicine and Preventive Medicine, Uppsala University, Uppsala, Sweden; 4 Department of Community Medicine and Rehabilitation, Physiotherapy, Umeå University, Umeå, Sweden

**Keywords:** COPD, pulmonary rehabilitation, primary care, survey study, access, health care professionals

## Abstract

**Background:**

Pulmonary rehabilitation (PR) is an important, evidence-based component for the management of individuals with chronic obstructive pulmonary disease (COPD). In daily practice, the majority of COPD patients are treated in primary care. However, information about the availability of PR in primary care in Sweden is lacking. The aim was to investigate the availability of rehabilitation resources in primary care settings for patients with COPD in Sweden.

**Methods:**

A cross-sectional descriptive design was applied, using web-based questionnaires sent to all primary care centres in four regions, comprising more than half of the 9.6 million inhabitants of Sweden. The main questionnaire included questions about the content and availability of rehabilitation resources for COPD patients. PR was defined as exercise training and one or more of the following activities: education, nutritional intervention, energy conservation techniques or psychosocial support.

**Results:**

A total of 381 (55.9%) of the 682 primary care centres answered the main questionnaire. In addition to physicians and nurses, availability of healthcare professionals for rehabilitation in primary care settings was physiotherapists 92.0%, occupational therapists 91.9%, dieticians 83.9% and social workers or psychologists 98.4%. At 23.7% of all centres, PR was not available to COPD patients – neither in primary care nor at hospitals.

**Conclusion:**

Despite high availability of professionals for rehabilitation in primary care settings, about one-quarter of managers at primary care centres stated that their COPD patients had no access to PR. This indicates a need to structure resources for rehabilitation and to present and communicate the available resources within the healthcare system.

Chronic obstructive pulmonary disease (COPD) is a global health problem with rising prevalence (1). Pulmonary rehabilitation (PR) is recognised as a core component of the management of individuals with COPD, and there is a need to increase the applicability and accessibility of PR in both primary and secondary care (2).

In the ATS/ERS statement on PR from 2013 (2), PR is considered to be an interdisciplinary intervention implemented by a dedicated, interdisciplinary team. Exercise training is considered to be the cornerstone of PR. In comparison with the statement from 2006 (3), a new goal of ‘effecting long-term health-enhancing behaviour change’ has been added. It is furthermore pointed out that PR at an earlier stage of disease has the potential to markedly alter the course of disease (2).

PR is also positioned within the concept of integrated care, defined by the WHO as ‘a concept bringing together inputs, delivery, management and organisation of services related to diagnosis, treatment, care, rehabilitation and health promotion’ (4).

In a recent survey, the content and organisational aspects of PR programmes were investigated to make an appraisal of their heterogeneity worldwide. It was concluded that there were large differences in all aspects that were surveyed (5). In a systematic review of PR in seven countries, it was reported that components provided in PR were similar between countries, while outcome measures demonstrated variation. Functional walk tests were the most common outcome measure, while scales measuring activities of daily living were minimally utilised. It was also observed that a very small number of patients were enrolled in PR (6).

In addition, it is known that patients with COPD are markedly inactive in daily life (7). Furthermore, reduction in physical activity starts early in the disease (8, 9), and many of the co-morbidities linked with COPD are associated with lack of physical activity (10). According to recent international guidelines for the management of COPD, physical activity is recommended to all patient categories (A–D: new assessment system) and PR is considered essential in groups B, C and D (1). Therefore, it is important that patients with COPD have the opportunity to participate in easily accessible physical activities and rehabilitation. As logistical aspects (transportation) are emphasised as being an important barrier (11–15), rehabilitation close to the patient's home would be preferable.

Primary care has the main responsibility for the management of patients with COPD in Sweden. In a Swedish study from 2014, one-third of the COPD patients in primary care had a forced expiratory volume in one second (FEV_1_) less than 50% predicted indicating severe disease (16). Despite the clear evidence that PR is important for patients with COPD, and the fact that most COPD patients are treated in primary care, no study has so far investigated the resources available for PR and exercise training in primary care. In Swedish primary care, asthma/COPD clinics are a common way of organising COPD care (17). The asthma/COPD clinics, led by a disease-specialist primary care nurse, have a specially appointed general practitioner as the responsible physician and are integrated in the primary healthcare centres. Other healthcare professionals such as physiotherapists and occupational therapists are often organised in separate units, but are often located in the same building. The population density in Sweden is low, especially in the northern part of the country, and it may be necessary to travel long distances to healthcare providers.

A survey of hospital-based PR in Sweden (15) showed that a very low proportion of patients with COPD took part in hospital-based PR programmes. It is therefore important to examine the availability of rehabilitation resources in primary care in Sweden.

The aim of this study was to investigate the availability and content of PR in primary care settings for patients with COPD in Sweden.

## Methods

### Design

The study had a cross-sectional, descriptive design. A first web-based main questionnaire was sent to the managers of all primary care centres in four regions, comprising 12 of the 20 county councils in Sweden. The four regions were selected to be representative of Sweden regarding demography and geographic areas. The catchment area included more than half (6.2 million) of the 9.6 million inhabitants in Sweden. The main questionnaire was sent in June 2012 and three reminder emails were sent in July, September and October 2012.

The main questionnaire, specific for this study, consisted of questions concerning the following: catchment area or number of registered patients per centre, public or private management, distance to a hospital with COPD clinic and whether there was an asthma/COPD clinic at the centre; furthermore, availability of professional caregivers (physiotherapist, especially for COPD patients, and occupational therapist, dietician, social worker or psychologist for patients in general): 1) at the centre, 2) at another primary care centre, 3) at another centre outside a hospital and not connected to a special primary care centre and 4) in a hospital-based unit. The last question was whether PR was available for COPD patients in the four sites, 1) to 4), during the year 2011. In this survey, PR was defined as exercise training and one or more of the following activities: education, nutritional intervention, energy conservation techniques or psychosocial support (3).

In February 2013, a web-based follow-up questionnaire, with three reminder emails, was sent to those who had answered that their COPD patients had access to PR during 2011. This questionnaire was used in a survey to all hospitals in Sweden, described by Wadell et al. (15).

A non-responder telephone survey with randomly selected centres was conducted among the centres that did not respond to the main questionnaire.

### Ethics

According to the regional ethics committee in Uppsala, Sweden, ethical approval was not required for this study. Data are presented in such a way that it is not possible to identify separate centres.

### Data analysis

Descriptive statistics, with data presented as frequency and percentage, and chi-square test, were used. In the analysis, resources in primary care were defined as all centres outside hospitals: sites 1) to 3).

## Results

### Main questionnaire

#### Response rate

**Fig. 1:**
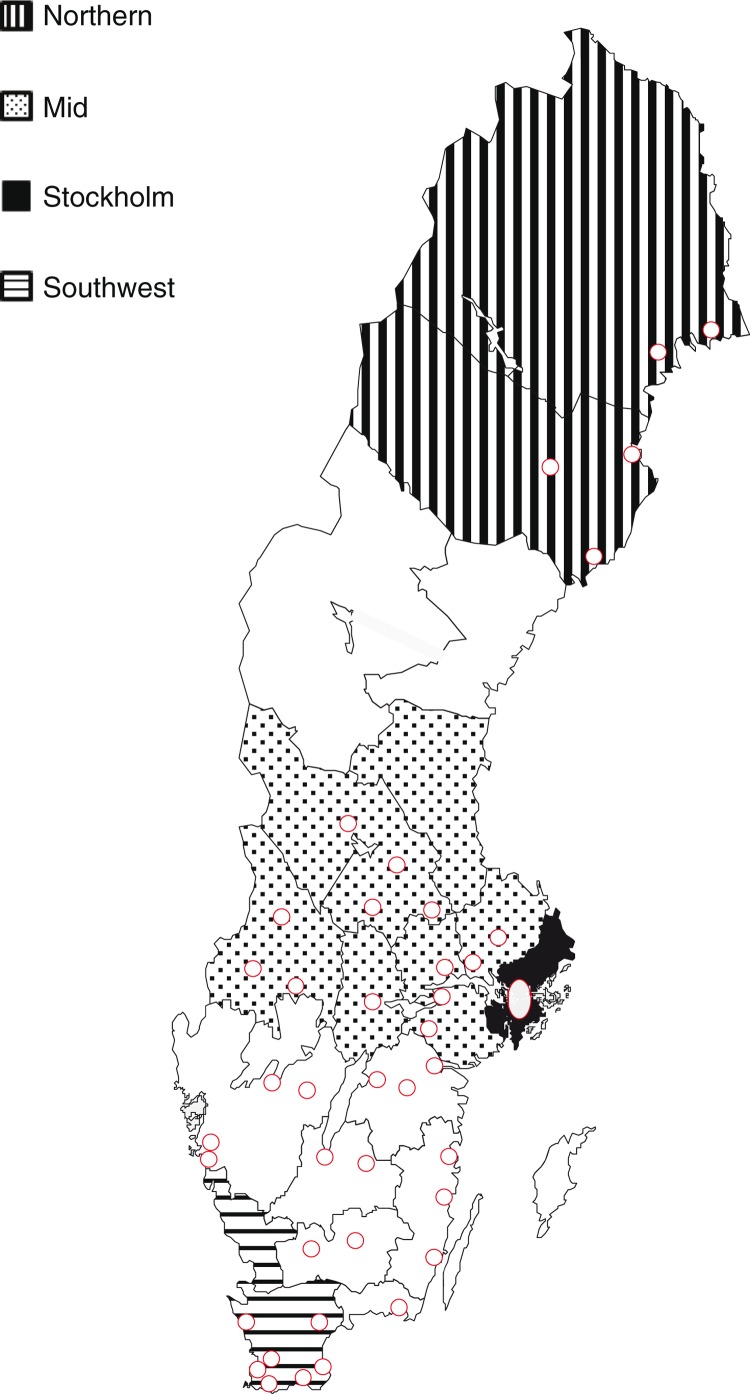
The four investigated regions in Sweden. Circles indicate hospitals with pulmonary rehabilitation according to Wadell et al. (15).

**Fig. 2:**
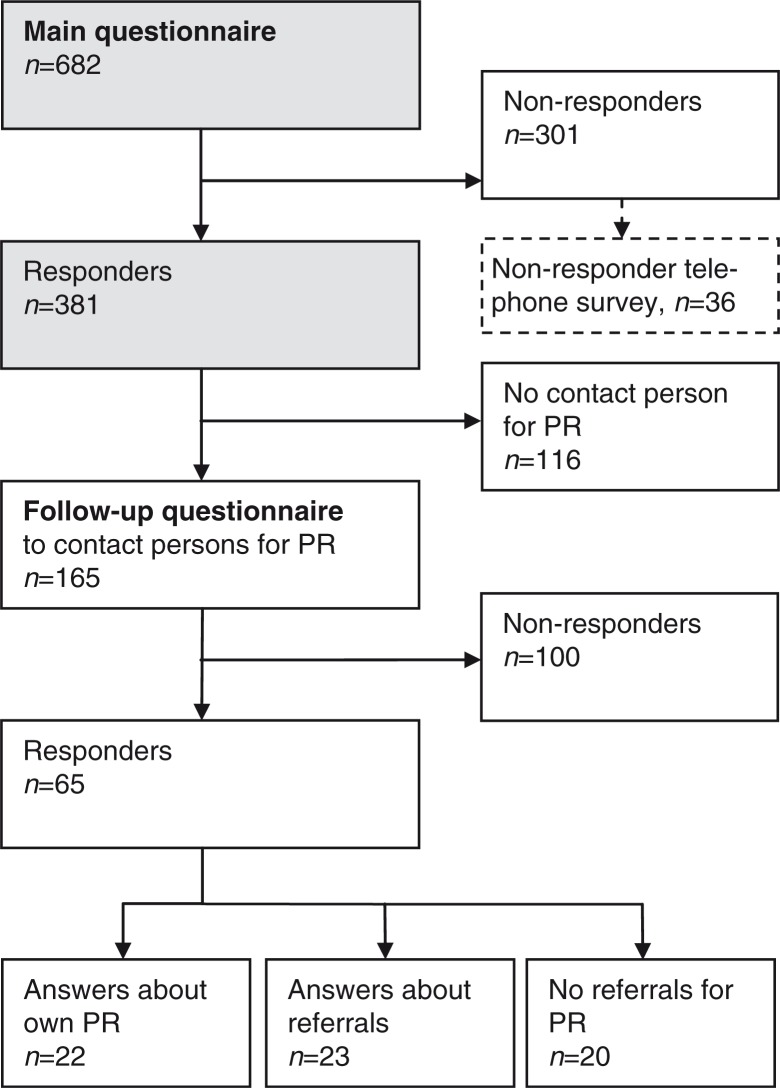
Flow chart of answers to main and follow-up questionnaire. PR, pulmonary rehabilitation.

In the four regions (Fig. 1), all 682 primary care centres were contacted, and managers from 381 centres (55.9%) responded to the main questionnaire (Fig. 2). As described in Table 1, there are great differences across Sweden between the four regions, regarding both demography and structure of healthcare resources.

**Table 1:** Demography data and hospitals with pulmonary rehabilitation in the four investigated regions in Sweden

Region	Population (1,000 inhabitants)	Population density (per km^2^)	Primary care centres	Responding centres and response rate	Hospitals with PR^a^	Distance (km) to hospital^b^, median (range)
Northern	509	3.3	77	40 (51.9%)	5	30.0 (0–350)
Mid-Sweden	1,982	21.7	229	144 (62.9%)	13	20.0 (0–170)
Stockholm	2,127	325.9	199	77 (38.7%)	6	10.0 (0–45)
Southwest	1,567	95.6	177	120 (67.8%)	7	7.5 (0–100)
All four regions	6,185	23.2	682	381 (55.9%)	31	12.0 (0–350)
Sweden	9,556	23.5	1,197		46	

PR, pulmonary rehabilitation.

a Hospitals according to Wadell et al. (15);

b hospital receiving referrals of COPD patients.

#### Healthcare professionals and rehabilitation

**Fig. 3:**
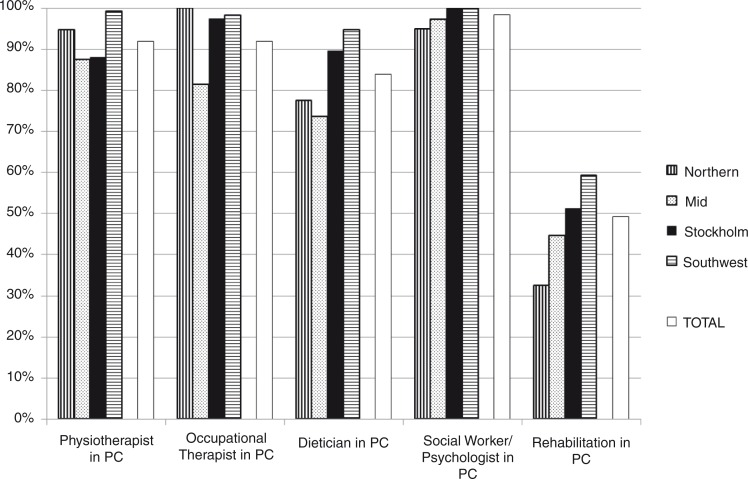
Availability of health care professionals and rehabilitation in primary care in four regions in Sweden (at the centre or another primary care centre or another centre outside hospital). PC, primary care.

In addition to physicians and nurses, the availability of healthcare professionals for rehabilitation in primary care settings was physiotherapists 92.0%, occupational therapists 91.9%, dieticians 83.9% and social workers or psychologists 98.4% (Fig. 3). There were some regional differences, as presented in Table 2, especially regarding access to rehabilitation in the primary care setting. Even with all professionals available and the existence of an asthma/COPD clinic, 42.6% of the centres nevertheless stated that there was no access to PR in primary care.

**Fig. 4:**
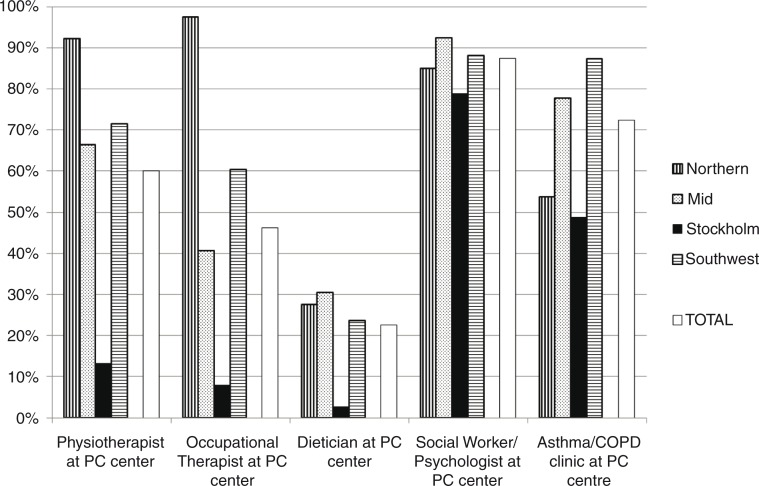
Availability of health care professionals and asthma/COPD clinic in primary care within their own centre in four regions in Sweden. PC, primary care.

Because of different organisation models, the availability of professionals within their own centres varied between the regions, from dieticians in 3% of the centres to occupational therapists in 98% of the centres (Fig. 4). Regarding access to PR for COPD patients, 49.3% of the centres stated they had access to primary care PR, 26.9% had access to hospital-based PR and 23.7% stated no access (Table 2).

**Table 2:** Location of pulmonary rehabilitation and asthma/COPD clinics in primary care in four regions in Sweden

Region	PR in PC; at their own centre (%)	PR anywhere in PC (%)	PR in hospital only (%)	No access to PR (%)	Asthma/COPD clinic in PC (%)
Northern (*n*=40)	10 (25.0)	13 (32.5)	15 (37.5)	12 (30.0)	21 (53.8)
Mid-Sweden (*n*=141)	50 (35.5)	63 (44.7)	46 (32.6)	32 (22.7)	108 (77.7)
Stockholm (*n*=76)	18 (23.7)	39 (51.3)	10 (13.2)	27 (35.5)	37 (48.7)
Southwest (*n*=118)	55 (46.6)	70 (59.3)	30 (25.4)	18 (15.3)	103 (87.3)
All four regions (*n*=375)	133 (35.5)	185 (49.3)	101 (26.9)	89 (23.7)	269 (72.3)

Six missing answers on the questions about location of PR. PR, pulmonary rehabilitation; PC, primary care.

#### Non-responders

The non-responder telephone survey (*n*=36) (Fig. 2) showed no difference (*p*>0.05) in answers to the main questionnaire compared with responders (*n*=381), except for PR, where 94.4% of non-responders stated that they had access to PR, compared with 76.3% of responders (*p*=0.022).

### Follow-up questionnaire

#### Response rate

The follow-up questionnaire was sent to the 165 centres where rehabilitation contact persons were identified, and answers were received from 65 (39%) centres.

#### Referrals

Forty-three centres answered only the initial three questions about referrals, because they did not have PR at the centre (Fig. 2). Twenty-three centres reported that they referred their patients to PR, 13 referred them to hospitals, five referred them to other units outside hospitals and five referred their patients to physiotherapists and other professionals working alone.

The remaining 22 centres had PR and answered the whole follow-up questionnaire.

#### Healthcare professionals and composition of the rehabilitation team

The most common compositions of professionals in the rehabilitation team were combinations of physiotherapist and nurse (15 centres) or physiotherapist and occupational therapist (15 centres). Physiotherapists were available in all 22 centres that answered the whole follow-up questionnaire. Seven centres reported that their teams consisted of physiotherapist, occupational therapist, nurse, dietician, physician, and social worker or psychologist.

#### Programme content

The main focus of all rehabilitation programmes was exercise training, consisting of components such as breathing exercises (20 of the 22 programmes), lower extremity resistance training (18/22), range of motion exercises (18/22), and aerobic exercise and cycling (16/22). Other training modalities used were group training on land (10/22) and in water (4/22).

Education was included in 16 of the 22 programmes (73%).

## Discussion

This survey described the availability of healthcare professionals and PR in primary care for COPD patients in Sweden. The availability of different healthcare professionals was high, but nevertheless 24% of the centres stated that their COPD patients had no access to PR. The main question was how to make individually tailored PR accessible to COPD patients.

Inactivity can have a detrimental effect on physical deconditioning and health-related quality of life. There are descriptions of COPD patients avoiding exercise and adopting a less active lifestyle early in the course of the disease. Reduced walking time has been reported in patients with GOLD stage II. Other events, such as exacerbations of the disease, further contribute to the adoption of an inactive lifestyle (18). The earlier the vicious circle of inactivity, deconditioning and social isolation can be interrupted, the better the outcome for the patient. Van Remoortel et al. concluded that reduction in physical activity starts early in the disease, especially in those with mild symptoms of dyspnoea, lower levels of diffusion capacity and exercise capacity (8). In primary care, there may be a substantial group of patients who could benefit from enhancing exercise capacity (19). Chavannes et al. pointed out that simpler rehabilitation programmes could be provided for less severe patients in primary care (20). This is supported by recent studies, where it is described that patients with mild COPD may benefit from PR, with positive effects on exercise capacity and quality of life; further studies are needed to confirm this (21, 22). Blackstock et al. suggested that PR based on exercise training, where multidisciplinary education cannot be offered, may be an option when resources are limited (23). A long-term physiotherapist-led PR programme, with a lower training intensity and frequency than currently recommended, has been used by Baumann et al. (24). In their study, clinical improvements were achieved in terms of exercise capabilities and health-related quality of life in patients with moderate to severe COPD, but further studies are needed to confirm this (24).

It is obvious that various levels of care are required, based on the individual's needs, as presented by Wagg (25) in a model including ‘integrated care’ and PR. This model is also referred to in the latest ATS/ERS statement on PR (2).

In a Cochrane review (26), the effects of integrated disease management programmes and interventions for patients with COPD are evaluated. These programmes consist of different components of care, in which different healthcare providers co-operate and collaborate to provide efficient and good quality care. Conclusions from the review are that integrated disease management programmes improve disease-specific quality of life and exercise capacity, and also reduce hospital admissions and hospital days per person (26). In primary care, the Dutch study by Chavannes et al. showed that a combination of optimal medical and non-medical treatments combined into a programme results in greater improvements in disease-specific quality of life compared with usual care (20).

In a Swedish survey in 2014 (the PRAXIS study) – answered by 893 randomly selected COPD patients, mean age 68 years, from 54 primary healthcare centres in the mid-Sweden region – only 7% of the COPD patients had been treated by a physiotherapist during the previous year because of COPD, although almost all of the centres in the region had access to a physiotherapist (27). The corresponding percentages for treatment by other professional groups were occupational therapists 2%, dieticians 5% and social workers or psychologists 1%. These figures indicate that despite guidelines, and the high availability of different healthcare professionals in primary care, utilisation of these professionals is very low. After data collection was performed in our study, new national guidelines for Sweden regarding asthma and COPD from the National Board of Health and Welfare were published (November 2015) (28). In these guidelines, there is a large focus on rehabilitation based on evidence, and this will hopefully give support to improve access to and delivery of PR for suitable patients as pointed out in a recent international policy statement (29).

There are both barriers and enablers for COPD patients to participate in PR (12, 14). It is pointed out that logistical aspects (transportation) seem to be an important barrier (13). The better the accessibility of exercise training and rehabilitation, the more patients could benefit from these resources. Therefore, rehabilitation in primary care settings that are also geographically close to the patients could overcome some barriers for the patients to participate. Even if not all resources for PR are available, it would nevertheless be important that the basic components of enhanced physical activity and basic exercise training could be made accessible and provided close to COPD patients.

The present study implies that the use of telehealthcare may be an option, especially in rural areas such as in the northern part of Sweden. A recent systematic review found improvements in physical activity level as a result of healthcare interventions, but the studies were heterogeneous and further studies are needed (30). Another study of nurse-initiated telephone follow-up has reported negative effects on health status and resource use in primary and secondary care (31). The field of telehealthcare is under investigation and will be further developed.

Limitations of the study include the facts that the main web-based survey was addressed and sent by email to the managers of the primary care centres, and that registers included small primary care centres; these two factors might have contributed to the low response rate. In this study, it was not possible to validate the answers further, and the main questionnaire was not always answered by the healthcare professionals directly engaged in PR. The follow-up questionnaire, where the aim was to describe the programmes, was only answered by a few centres. Nevertheless, these answers state that components similar to those included in hospital-based rehabilitation were used, although there is heterogeneity between programmes. This is in accordance with the global investigation by Spruit et al. (5).

## Conclusions

This study shows high availability of healthcare professionals for rehabilitation in primary care settings in Sweden. Nevertheless, about one-quarter of managers in primary care centres stated that their patients with COPD did not have access to PR – not even hospital-based. This indicates a need to structure resources for rehabilitation and to present and communicate the resources available. It is also necessary to increase awareness regarding the importance of PR in primary care.
